# An ethnobotanical study of the less known wild edible figs (genus *Ficus*) native to Xishuangbanna, Southwest China

**DOI:** 10.1186/1746-4269-10-68

**Published:** 2014-09-24

**Authors:** Yinxian Shi, Huabin Hu, Youkai Xu, Aizhong Liu

**Affiliations:** Key Laboratory of Economic Plants and Biotechnology, Kunming Institute of Botany, Chinese Academy of Sciences, 650201 Kunming, P R China; Key Laboratory of Tropical Plant Resources and Sustainable Use, Xishuangbanna Tropical Botanical Garden, Chinese Academy of Sciences, 666303 Xishuangbanna, Yunnan P R China

**Keywords:** *Ficus*, Ethnobotany, Management, Fruit, Vegetables, Xishuangbanna

## Abstract

**Background:**

The genus *Ficus*, collectively known as figs, is a key component of tropical forests and is well known for its ethnobotanical importance. In recent decades an increasing number of studies have shown the indigenous knowledge about wild edible *Ficus* species and their culinary or medicinal value. However, rather little is known about the role of these species in rural livelihoods, because of both species and cultural diversity.

**Methods:**

In this study we 1) collected the species and ethnic names of wild edible *Ficus* exploited by four cultural groups in Xishuangbanna, Southwest China, and 2) recorded the collection activities and modes of consumption through semi-structured interviews, 3) investigated the resource management by a statistical survey of their field distribution and cultivation, and 4) compared and estimated the usage intensities by the grading method.

**Results:**

The young leaves, leaf buds and young or ripe syconia of 13 *Ficus* species or varieties are traditionally consumed. All the species had fixed and usually food-related ethnic names. All four cultural groups are experienced in the collection and use of edible *Ficus* species as vegetables, fruits or beverages, with the surplus sold for cash income. Different cultural groups use the *Ficus* species at different intensities because of differences in availability, forest dependency and cultural factors. Both the mountain and basin villagers make an effort to realize sustainable collection and meet their own and market needs by resource management *in situ* or cultivation.

**Conclusions:**

In comparison with reports from other parts of the world, ethnic groups in Xishuangbanna exploited more edible *Ficus* species for young leaves or leaf buds. Most of the edible species undergo a gradient of management intensities following a gradient of manipulation from simple field gathering to *ex situ* cultivation. This study contributes to our understanding of the origins and diffusion of the knowledge of perception, application and managing a group of particular plant species, and how the local culture, economic and geographical factors influence the process.

## Background

The genus *Ficus* consists of over 800 species of trees, shrubs, vines and epiphytes in the family Moraceae, which have a wide distribution and multiple uses in most tropical and subtropical regions throughout the world. They are traditionally used as medicine or food plants, ornamental trees, religious plants, lacca hosts, fodder, fuel wood, hedges or enclosures. The importance of *Ficus* as a global spiritual and material resource for humans has been well-documented [1, 2].

Some *Ficus* species are reported to be among the oldest human food sources. Fossil evidence suggests that the common fig (*Ficus carica* L.) has been cultivated for over 11,000 years, possibly predating cereal grains [3], and thousands of cultivars of this species have been developed worldwide. The biblical sycamore fig (*F. sycomorus* L.) is another old human food source and one of the largest fruit producers. It was identified as a key food source for Pliocene hominids along the Baragoi River in Nachola and Baragoi, in northern Kenya [4]. Nowadays the most well-known species is *F. carica*, which produces the commercial common fig (fig is also the general name of the “fruit” of the genus). Specifically, the common fig is the syconium of *F. carica* var. *domestica*, which is functionally a female individual of *F. carica*. However, it was only in recent decades that it became known how many more culinary uses the genus *Ficus* can provide beyond their nutritious raw syconia. A growing number of studies have noted the significance of the edible *Ficus* species as vegetables [5–9], for tea or beverage preparation [10–12], for jelly and jam production [13] or for medicinal purposes [14–17].

Our previous ethnobotanical survey conducted in southwest Yunnan, China, found that *Ficus* was the most frequently consumed genus (with 8 species) of the 220 plant genera which contain species used as wild vegetables [6]. *Ficus* was also the most frequently used genus (7 species) among the 82 genera containing wild fruits in the region [18]. Specialized studies of these wild edible *Ficus* species are needed to clarify their ethnobotany, including vernacular names, modes of consumption, availability and management, and possible multiple uses. Since most of the species identified in these surveys are native to the Xishuangbanna Dai Autonomous Prefecture, a cultural and biodiversity hotspot in southwest Yunnan, China, a research project was initiated to estimate the current and historic usage of edible *Ficus* species in Xishuangbanna, not only for their economic benefits, but also for their cultural richness and their roles in the long history of rural livelihoods.

## Methods

### Study area

Xishuangbanna (XSBN) is in the south of Yunnan province, southwest China, bordering Myanmar and Laos. It has an area of almost 20,000 km^2^ and is biogeographically located at the transitional zone with tropical Southeast Asia to the South, subtropical East Asia to the north, the Sino-Japanese floristic region to the east and the Sino-Himalayan floristic region to the west [19]. There are three seasons: a cool and foggy winter (November–February), a warm and dry ‘summer’ (March–April), and a warm and wet monsoon season (May–October). The region possesses an exceptionally diverse flora and 13 indigenous cultural groups have well-established ethnobotanical knowledge. *Ficus* is the most species-rich genus in the region [19], with 69 species, subspecies or varieties native to the area [20]. Because of their close relationship with natural forests, all the native culture groups in XSBN have developed knowledge systems related to the use and conservation of natural resources. Wild collection is traditionally an important subsistence activity. Four ethnic villages near natural reserves inhabited by Dai, Hani, Jinuo and Yao people were selected as sample sites (Table 1). The exact locations of the sample sites are withheld to protect the anonymity of the interviewees.). For each village, more than 95% of the villagers are indigenous. Relevant local markets were also visited.

**Table 1:** **Information on the ethnic villages sampled in Xishuangbanna, SW China**

Village	Altitude	Culture group	Population	Main income sources
Bakaxiaozhai	650 m	Jinuo	280	Rubber trees, wild collection
Dazhai	900 m	Yao	989	Cash crops (bean and corn), livestock, wild collection and hunting
Manlun	570 m	Dai	520	Rubber trees, farmland rent, part – time jobs
Nanbang	910 m	Hani	263	Cash crops (*Amomum villosum*, banana, etc.), livestock, wild collection and hunting

### Ethnobotanical data collection

Semi-structured interviews were conducted with 10–27 key informants in each village in March–October 2012, for details about the wild edible *Ficus* species, including their local nomenclatures, collection techniques and modes of consumption. The number of interviewees selected in each village mainly depended on the integrity and uniformity of information we obtained. Only those who could give details independently and according to their own experience, or could give a practical demonstration, were counted as key informants. Interviewees were first shown a group of sample species (photographs or fresh specimens) that were expected to be used. Species mentioned by informants that were not included in the samples were given special attention as new records. The Yao villagers living in Dazhai are monolingual, with only the village school students and staff able to speak Mandarin, so our interviews were conducted in the school with Yao students and teachers. Dai, Hani and Jinuo interviews were conducted in the villagers’ homes, farming plots or collection sites using Mandarin or their own language, with the help of local field assistants.

Field investigations were designed to assess the availability of edible fig resources, including their field distribution and home cultivation. Since the mountain villages (Bakaxiaozhai, Dazhai and Nanbang) do not have private homegardens, they consume only wild *Ficus*. We chose Nanbang as a representative mountain village to assess the distribution of the fig individuals they use. Six key informants were asked to guide field travel in the area surrounding their village in March 2012. *Ficus* individuals in dietary use and within one-hour walking distance (along twisting narrow footpaths in five directions) from the village site were recorded, including their species names, the numbers of plants, and their diameters at breast height (DBH). We chose “one-hour walking distance” to include most plots where the daily collection activities happen and the distance was accessible for all of our local guides. The Dai, who settle in valleys, basins or on river banks at lower elevations, are the major culture group in XSBN and have developed diverse homegardens. Most *Ficus* parts they eat come from these or from around their courtyards. We visited 107 households in Manlun village in May–June 2012 to collect data regarding the species, numbers of plants, and DBH of all edible *Ficus* species cultivated.

Observations of trading in *Ficus* products were conducted in three local markets related to the selected villages. They are the farm products market of 1) Menglun township, 2.2 km to Manlun and 5.7 km to Bakaxiaozhai, 2) Mengla township, 31 km to Nanbang, and 3) Jinghong city, which assemble farm products and wild collections from multiple ethnic groups and wide areas. All the villages can access their markets by motor vehicles. We selected Menglun market for long-term (November 2013 to April 2014) monitoring to collect basic data (species, price, sources, etc.) and determine the amounts sold and peak season, because the market has the most diverse *Ficus* products and modest size. We did market survey in Mengla and Jinghong during peak season to assess the universality of the trading activities. Only part of the result is shown in this study. More details on the amounts sold and their sources will be included in another publication because it needed additional investigations.

### Data analysis

Based on the usage intensity classification for wild fruits of Chen et al. [18], we defined five levels of usage intensity according to our work described above. The five levels are coded as 1) **–**, no usage record; 2) **+**, consumed occasionally or just by children and hunters; 3) **+ +**, frequently consumed and often gathered regularly during harvest season; 4) **+ + +**, frequently consumed and the surplus may be sold in the local market or processed for out-of-season use, sources protected; 5) **+ + + +**, cultivated in homegardens for consumption and sale.

All voucher specimens collected in our field work were deposited in the herbarium of the Xishuangbanna Tropical Botanical Garden (HITBC), Chinese Academy of Sciences.

## Results

### Edible species and their ethnic nomenclature

Thirteen edible *Ficus* species or varieties and their corresponding local names were recorded. For *F. maclellandii* var. *rhododendrifolia* and *F. vasculosa*, the details were collected from the local markets. For the other 11 species, information was obtained by interviews of key informants in the four sample villages. The pronunciations of the Mandarin and ethnic names were recorded as accurately as possible in the Chinese phonetic alphabet (Table 2).

**Table 2:** **Wild edible** ***Ficus*** **species used by each ethnic group and their names**

Species ^A^	Mandarin name ^B^	Ethnic name ^C^				Specimen number
		Dai	Hani	Jinuo	Yao	
*F. altissima* Bl.	Da qing shu (lofty tree)	——	niza nihao	paleng*, palu*	——	S120315003
*F. auriculata* Lour. (*F. roxburghii* Wall.)	Xiang er rong, Mu gua rong (elephant ear fig or papaya fig)	pàk wā	xibu(bu) ma	sipu	nuoge biu	S140227022
*F. callosa* Willd.	Ying pi rong (hard skin fig)	pàk dédá	——	paleng	——	S130911018
*F. hirta* Vahl.	Wu zhi mao tao, Cu ye rong (five – finger hairy fig or rough leaf fig)	——	acalama axi	Wamo dousou	bulana biu	S121003006
*F. maclellandii* King var. *Rhododendrifolia* Corner	Du juan ye rong (azalea leaf fig)	pàk yī	——	——	——	S140415024
*F. oligodon* Miq.	Ping guo rong (apple fig)	pàk wā	xibu qi	sipu	nuoge zam biu	S140404023
*F. racemosa* L. (*F. glomerata* Roxb.)	Ju guo rong, Ma lang guo (Cluster fig)	pàk dē	tang bule	guole, mulu se*	——	S140417026
*F. semicordata* B.-H. ex Sm.	Ji su guo (chicken crop fig)	mūnā	xigugu ma	sigeiyao	mule lum biu	S121028007
*F. semicordata* var. *montana* Amatya	Ji su guo (Small fruit variety of chicken crop fig)	mūnā	xigu misi	sigeiyaogeiyao mi	mule zam biu	S120313002
*F. tikoua* Bur.	Di ban teng, Di shi liu (ground pomegranate)	——	——	——	muduoniang	S121029009
*F. vasculosa* Wall. ex Miq.	Tu mai rong, Shan tian cai (sweet mountain greens)	pàk dé gái	——	——	——	S120518004
*F. virens* Ait.	Lv huang ge shu, Suan bao shu (sour buds tree)	pàk yī	niza bao	neme adao	gelong den	S131201021
*F. virens* Ait. var. *sublanceolata* (Miq.) Corner	Huang ge shu, Suan bao shu (sour buds tree)	pàk yī	——	neme adao	——	S130901016

^A^Botanical names in brackets are frequently seen synonyms.

^B^Name in brackets is English equivalent of the Mandarin name.

^C^“——” means that the species is not reported as edible by the ethnic group.

*Quote according to Wang and Long [21].

In the Dai language, names of wild plants used as vegetables are often preceded by “pàk”. The words “liāng” (reddish), “xiū” (green), “háo” (white), “núi” (smaller) and “lōng” (bigger) are often added to discriminate the edible parts of similar fig plants. Some *Ficus* forms distinguished by local people are apparently not yet recognized taxonomically. Sometimes, the flavor is reflected in the plant name. For example, the Dai word for chicken, “gái.” is used in the Dai name for *F. vasculosa*, describing the chicken-soup like taste of the young leaves used in vegetable soup.

In the Hani language, “xibu” refers to the fig fruits borne in clusters on the trunk or thick leafless branches, while “xigu” refers to fruits borne in long and slender leafless braches from the trunk and near the ground. The words “ha” (bitter, here means inedible male figs), “ma” (bigger) and “qi” or “misi” (smaller) are often added after the formal name. The Hani word “niza” means a guest who stayed too long and can’t be driven away, and is used for strangling fig trees! Typical strangler species, such as *F. virens*, are disliked by the Hani even though they know the leaf buds are edible.

In the Jinuo language, “neme” refers to leaf buds with a sour taste and “adao” is the common name for leaves. In the Yao language, “nuoge” is a common name for edible plants with white latex. The words “lumu” and “zamu” refer to “bigger” and “smaller” fig fruits. “Biu” is a common name for edible fruits, and “den” means young leaves or leaf buds.

### Collection and form of consumption

**Figure 1:**
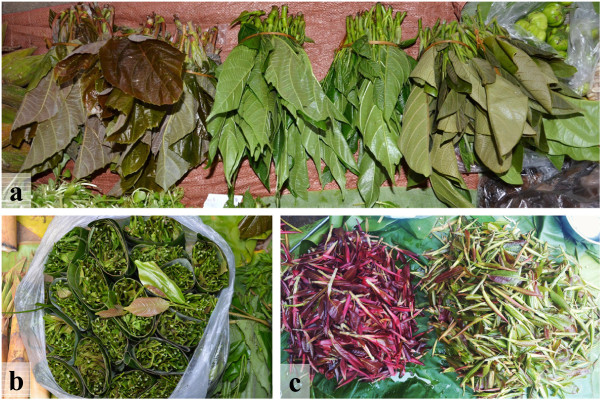
**Some examples of** ***Ficus*** **vegetables sold in local markets in Xishuangbanna, SW China.** From left to right: young leaves of *F. auriculata*, *F. racemosa* and *F. callosa* tied using bamboo sliver **(a)**; Young leaves of *F. vasculosa* wrapped in leaf slices of genus *Musa* L. **(b)**; Piles of red and green leaf buds of *F. virens* on the leaves of genus *Musa* L. **(c)**.

All four culture groups use the young or ripe female figs and young leaves or leaf buds of multiple *Ficus* species as fruits, beverages or vegetables, with only minor differences in modes of consumption. Surplus young female figs of *F. auriculata* and young leaves or buds of six species are sold for cash income in local markets (Figure 1). Generally, the appropriate harvest season for young leaves or buds is late winter to summer and for fruits the monsoon season (Table 3), but there are exceptions to this timing under both natural conditions and with human intervention, such as frequent pruning to prolong the crop life of young leaves or buds.

**Table 3:** **The growth habit, habitat, harvest season and consumption form of the** ***Ficus*** **found in the ethnic diet in Xishuangbanna, SW China**

Species ^A^	Growth habit (M/D) ^B^	Habitat	Intensive harvest period	Edible parts	Mode of consumption ^C^
FAL	Large trees (M)	Insides and margins of the thick forest in valleys or mountains, or grow as individuals in plains	February – March	Leaf buds	Vegetables boiled with pork ribs (water blanching before cook, Hani), the stipules act as sour taste ingredients
FAU	Small trees (D)	Tropical or subtropical forests in moist valleys, or surroundings of farmland and village	January – April (young leaves), March – July (ripe female figs)	Young or ripe female figs, young leaves	Ripe figs are eaten raw or for making jelly beverage; Young figs are used as salad with condiments (Dai and Jinuo); Young leaves are used as vegetables boiled with spareribs (water blanching or rubbing with salt before cook); Young figs and young leaves are sold for cash income
FCA	Large trees (M)	Forests in basins or valleys in lower mountains	January – May	Young leaves	Vegetables cooked with tomato (the red kind need water blanching before cook, Dai, Jinuo); sold for cash income
FHI	Shrubs or small trees (D)	Slopes or margins of mountain forests or open fields near villages	August – October	Ripe female figs	Child snacks (Hani, Jinuo and Yao)
FMR	trees (M)	Plains or thin forests along river and stream sides	February – April	Leaf buds	Fresh vegetables, or store up after quickly baked and dried for use out of season (Dai); Being sold for cash income
FOL	Small trees (D)	Forests in higher mountainous areas,	(Similar to FAU)	Ripe female figs, young leaves	Ripe figs are eaten raw or for making jelly beverage; Young leaves are used as vegetables boiled with spareribs (water blanching or rubbing with salt before cook); Young leaves are sold for cash income
FRA	Large trees (M)	Thin forests along river and stream sides, or valleys of lower mountains	March – May	Young leaves	As salad with condiments or as vegetables cooked with green moss (Dai, Hani, Jinuo)
FSE	Small trees (D)	Forest edge or thin forests in valleys, beside rivers and roads	Irregular (2 – 3 crops per year)	Ripe female figs	Ripe figs are eaten raw
FSM	Small trees (D)	Forest edge or road side	Irregular (3 crops per year)	Ripe female figs	Ripe figs are eaten raw
FTI	Prostrate woody vines (D)	Slopes of limestone mountain and grass land at higher elevations	June – September (ripe female figs)	Ripe female figs; whole plant	Ripe female figs are eaten raw; Whole plant is used for tea preparation (Yao)
FVA	Trees (M)	Seasonal rain forests at lower elevations	January – June	Young leaves	Soup vegetables boiled with other wild greens or fried vegetables (Dai, Hani); Sold for cash income
FVI	Large trees (M)	Forests in valleys or lower mountains	January – April	Leaf buds	As salad with condiments (Yao) or as vegetables boiled with pig trotter, the stipules give a sour taste; Being sold for cash income
FVS	Large trees (M)	Forests in valleys or lower mountains, or growing as individuals in plains	January – April	Leaf buds	Vegetables boiled with pig trotter, the stipules give a sour taste (Dai)

^A^FAL, *F. altissima*; FAU, *F. auriculata*; FCA, *F. callosa*; FHI, *F. hirta*; FMR, *F. maclellandii* var. *rhododendrifolia*; FOL, *F. oligodon*; FRA, *F. racemosa*; FSE, *F. semicordata*; FSM, *F. semicordata* var. *montana*; FTI, *F. tikoua*; FVA, *F. vasculosa*; FVI, *F. virens*; FVS, *F. virens* var. *sublanceolata*.

^B^D, Dioecious; M, Monoecious.

^C^The consumption modes without brackets indication mean they are common to all four ethnic groups in this study.

### Resource management

**Figure 2:**
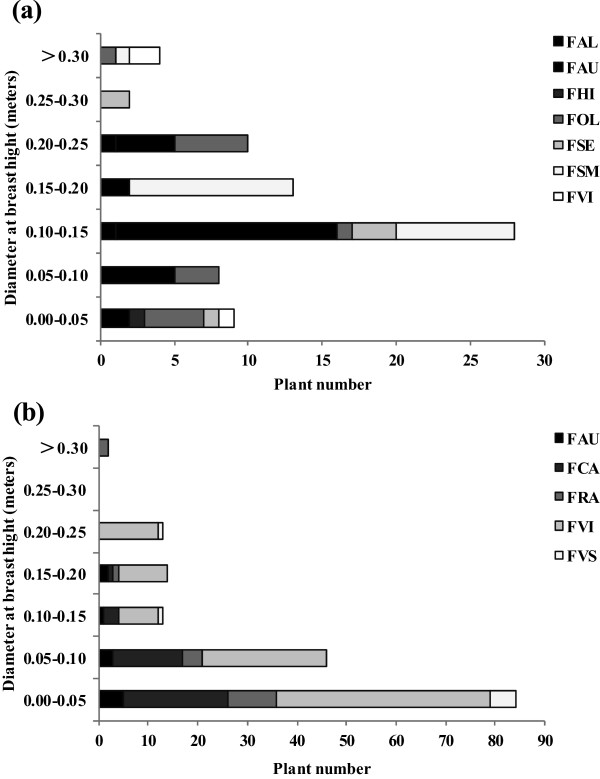
**The number and diameter at breast height (DBH) of wild edible** ***Ficus*** **individuals around Nanbang (a); The number and DBH of the edible** ***Ficus*** **individuals kept by households in Manlun (b). FAL**, *F. altissima*; **FAU**, *F. auriculata*; **FHI**, *F. hirta*; **FOL**, *F. oligodon*; **FRA**, *F. racemosa*; **FSE**, *F. semicordata*; **FSM**, *F. semicordata* var. *montana*; **FVI**, *F. virens*; **FVS**, *F. virens* var. *sublanceolata*.

Mountain villagers in Nanbang retain and protect the wild edible *Ficus* trees growing around the village *in situ*, and sometimes transplant individuals away from new farmland or road construction sites. Seventy four individuals of 7 species or varieties growing within one-hour walking distance from the village were recorded, mostly with a DBH of 0.10-0.15 m (Figure 2a). The villagers increase the production of young leaves and leaf buds by pruning during or out of the harvest season.

**Figure 3:**
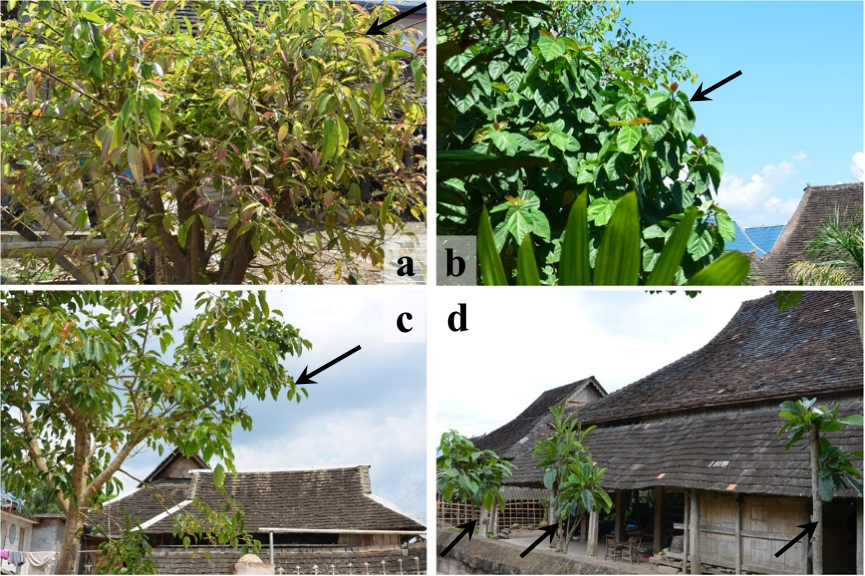
**Some examples of edible** ***Ficus*** **species cultivated by the Dai people in Xishuangbanna, SW China.** *F. maclellandii* var. *rhododendrifolia* cultivated in kitchen garden **(a)**; *F. auriculata* cultivated in homegarden **(b)**; *F. virens* planted around the courtyard **(c)**; Three individuals of *F. callosa* planted in the courtyard **(d)**. (Marked with black arrows).

In the basin village of Manlun, 4 edible *Ficus* species and 1 variety were cultivated and 70 households (65% of the total) had them. The trees were grown in kitchen gardens or in and surrounding the home yard (Figure 3b, c, d). They were grown from branch cuttings or from seedlings transplanted from nature, except that most *F. racemosa* and a few *F. virens* individuals grew spontaneously from seed. The number of *Ficus* individuals owned by each household range from 1 to 9 (Table 4). Most are young trees and their DBH is less than 0.1 m (Figure 2b). The other two cultivated species, *F. maclellandii* var. *rhododendrifolia* and *F. vasculosa*, were not found in Manlun but in two other Dai villages according to the people who sold them in the market. Field surveys showed that *F. maclellandii* var. *rhododendrifolia* was planted as individuals by some households (Figure 3a), while *F. vasculosa* was planted in groups of small trees that can be managed and picked like tea (see lower part of the photo in personal cover page), so they are also known as “tea-leaf vegetables”.

**Table 4:** **The number and location of edible** ***Ficus*** **species kept by households in Manlun village in Xishuangbanna, SW China**

Species	Total number of plants	Location ^A^			Percent of households with each species ^B^	Number of plants kept by each household	
		IC	SC	KG		Average	Maximum
*F. auriculata*	11	8	0	3	15.7%	1.0	1
*F. callosa*	39	17	1	21	31.4%	1.8	4
*F. racemosa*	17	5	7	5	21.4%	1.1	2
*F. virens*	98	49	11	38	82.9%	1.7	9
*F. virens* var. *sublanceolata*.	7	0	0	7	5.7%	1.8	4

^A^IC, in courtyard; SC, surrouding courtyard; KG, in kitchen garden.

^B^A total of 70 households were recorded keeping edible *Ficus* species.

### Comparative evaluation of the usage intensities and preference

Among the 13 edible *Ficus*, five species (*F. auriculata*, *F. oligodon*, *F. semicordata*, *F. semicordata* var. *montana* and *F. virens*) are common to all four culture groups. Only two of these (*F. auriculata* and *F. virens*), however, are cultivated for home and commercial use, and reached the highest usage intensities (+ + + +). The other four “+ + + +” species (*F. callosa*, *F. maclellandii* var. *rhododendrifolia*, *F. vasculosa* and *F. virens* var. *sublanceolata*) are mainly used by Dai people (Table 5).

**Table 5:** **Comparison of the usage intensities of each edible** ***Ficus*** **species among different culture groups**

Culture groups	Species *												
	FAL	FAU	FCA	FHI	FMR	FOL	FRA	FSE	FSM	FTI	FVA	FVI	FVS
Dai	–	+ + + +	+ + + +	–	+ + + +	+ + +	+ ++	+ +	+	–	+ + + +	+ + + +	+ + + +
Hani	+	+ + +	–	+	–	+ +	+	+ +	+ +	–	–	–	+
Jinuo	–	+ + +	+ + +	+	–	+ + +	+	+ +	+ +	–	–	+ + +	+ + +
Yao	–	+ +	–	+	–	+ +	–	+	+	+ +	–	+	+

* –, no usage record; +, consumed occasionally or just by children and hunters; + +, frequently consumed and often gathered regularly during harvest season; + + +, frequently consumed and the surplus amount may be sold in the local market or processed for out-of-season use, the resources are protected intentionally; + + + +, introduced or cultivated in homegarden for the convenience of daily use and commercial purpose. FAL, *F. altissima*; FAU, *F. auriculata*; FCA, *F. callosa*; FHI, *F. hirta*; FMR, *F. maclellandii* var. *rhododendrifolia*; FOL, *F. oligodon*; FRA, *F. racemosa*; FSE, *F. semicordata*; FSM, *F. semicordata* var. *montana*; FTI, *F. tikoua*; FVA, *F. vasculosa*; FVI, *F. virens*; FVS, *F. virens* var. *sublanceolata*.

## Discussion

### Comparison of the wild edible *Ficus*species uses among the four culture groups

The Dai, Hani, Jinuo and Yao represent four distinctly different cultural groups with different languages in XSBN. All four groups have accumulated extensive knowledge on using the female fruits (For dioecious fig taxa, only the female fruits are edible, they are seed producers and are larger in size, rich in nutrient and have a pleasant taste, while the male fruits function in breeding pollinator wasps are poor in nutrient and not palatable. For monoecious fig taxa, their fruits are the same and avoided by people because small size, poor taste and the presence of wasps), young leaves or leaf buds of *Ficus* species as food resources. The common consumed species across the four groups are *F. auriculata*, *F. oligodon*, *F. semicordata*, *F. semicordata* var. *montana* and *F. virens*. The wide consumption and sustained marketing of these species show that they are widely accepted food resources rather than a narrow cultural preference. All the species had fixed and usually food-related ethnic names, which indicates the long history of indigenous consumption.

Different cultural groups use the wild edible *Ficus* species to different extents, depending on their availability, the group’s dependence on forest resources, and cultural factors. Generally, the Dai people use them at the highest intensity because (i) they have been permanent inhabitants in XSBN for centuries, and have established homegardens for small-scale wild food or medicinal plant cultivation or domestication, and (ii) they live in basins and have better access to markets. The other three mountain culture groups have more recently established permanent living spaces in the region and do not have a culture of homegarden cultivation, depending more on forest collection. Our data show that the Hani people in Nanbang prefer fruits of *F. auriculata* and *F. oligodon* (“Xibu”). One of their orally transmitted legends tells that the first Hani people were born in a “Xibu” and survived by sucking the “milk” in it (the milky latex that characterizes figs). In addition, their ethnic epic records that “Xibu” is one of the first edible fruit plants they discovered in the forest during hunting activities. The villagers do not like strangling figs, such as *F. alltissima* and *F. virens*, since they often encounter huge strangling figs in the forest which have killed their host trees, and believe this is an omen of disaster. They therefore remove most of spontaneous seedlings around the village.

### The significance of the cultivation and commercialization of the wild edible *Ficus*species

The possibility of a wild plant resource continuing to meet both subsistence and market demands largely depends upon sustainable harvest by appropriate management, and the domestication of wild resources is crucial for resource management [22]. Domestication of common fig is thought to have started in the Mediterranean region in Early Neolithic period [3] and then spread worldwide. In XSBN, the Hani, Jinuo and Yao protect wild edible *Ficus* species while the Dai cultivate them. Except for the species traditionally cultivated in or around courtyards for ornamental and consumption purpose (such as *F. auriculata*), the cultivation of most *Ficus* species was triggered by the expansion of rubber plantations and resulting sharp decline in wild resource accessibility since the early 1980s. The indigenous villagers prefer to plant these species for their wide adaptability, high productivity, easy management and pleasant palatability. *Ficus* species are cultivated as tree fodder in many parts of the Himalaya region, with *F. auriculata* the most widely used species. Its fodder quality is far superior to paddy straw [23]. In the Bamileke region of Cameroon, *Ficus* species are propagated by pole cuttings and are an important part of agrarian system management [24]. These examples show that wild *Ficus* species undergo a gradient of management intensities from simple gathering, to nonselective incipient management, selective incipient management and occasionally *ex situ* cultivation [25]. But whether or not the incipient domestication has occurred is unknown.

The commercialization of wild edible *Ficus* species in XSBN is an important source of cash income for indigenous villagers. The leaves, buds and fruits of several *Ficus* species are sold in local markets and the whole plants are sold as ornamental trees. In other regions, however, only in north and central Vietnam are the near-ripe peeled or unpeeled fruits and young leaves of elephant figs (*F. auriculata* Lour. or *F. oligodon* Miq.) on sale in the market, and in Papua New Guinea the young leaves of dinner-plate figs (*F. dammaropsis* Diels.) are commonly sold in highland markets [8]. As Sawian et al. [26] reported, these products seldom appear to contribute a large share of a household’s total income generation, but are often important in bridging seasonal or other cash flow gaps.

### The characteristics of the wild edible *Ficus*species in Xishuangbanna compared to those in other regions

The use of wild *Ficus* species as food is widespread in areas where the genus occurs, especially in the Himalaya region, which is floristically similar to our study area. For the 13 edible *Ficus* species in this study, the consumption of 6 species has either not been reported from elsewhere (*F. altissima*, *F. callosa*, *F. maclellandii* var. *rhododendrifolia* and *F. vasculosa*), or only from South China (*F. tikoua* and *F. virens* var. *sublanceolata*). Consumption of the other 7 species had been reported from Nepal [7, 27] and some were reported as eaten in India [9, 13, 28, 29], North Laos [18], Vietnam [8] or Pakistan [16, 30].

No other region appears to consume the diversity of wild *Ficus* species eaten in XSBN. Moreover, in the species consumed only in XSBN as well as those used most intensively, leaves and buds are the major parts consumed. These are concentrated sources of vitamin E, vitamin B_1_ (thiamin), vitamin B_2_ (riboflavin), protein and minerals [31], and are apparently also rich sources of naturally occurred antioxidants [32], suggesting that they may make a significant contribution to the health and well-being of the consumers. Antioxidant potential has also been demonstrated in some other *Ficus* leaf samples [33].

## Conclusions

Our studies of wild, managed *in situ* and cultivated edible *Ficus* populations showed that their edible products are highly appreciated by the indigenous people of Xishuangbanna. We found that in both mountain villages, which have forest access but are far from markets, and basin villages, which have market access but no forests, figs are used as vegetables, fruits or beverages. Moreover, people in both situations invest effort in promoting the use of these species through artificial management. The Dai people who live in basin villages cultivate preferred species in and around their kitchen gardens and courtyards, encouraging intensive usage, with the possibility of artificial selection of plants with preferred characteristics. Further studies should be conducted to determine if these species are or can be ongoing incipient domestication. Finally, this study of a small geographic area suggests that the genus *Ficus* represents a largely untapped source of potential food resources for tropical people.
